# The neuroanatomy and connectivity of the ventral striatum and ventral capsule area in the Göttingen minipig

**DOI:** 10.1007/s00429-026-03115-7

**Published:** 2026-05-14

**Authors:** Anna Sofie R. B. Poulsen, Hamed Zaer, Bjarke Søgaard, Andreas N. Glud, Jens Christian H. Sørensen, Dariusz Orlowski

**Affiliations:** 1 Center for Experimental Neuroscience, Department of Neurosurgery, Aarhus University Hospital, Aarhus, Denmark; 2 Department of Clinical Medicine, Aarhus University, Aarhus, Denmark

**Keywords:** Göttingen minipig, Ventral striatum, Neuronal tracing, Neuroanatomy, Nucleus accumbens

## Abstract

A considerable proportion of patients with major depressive disorder (MDD) do not respond to current treatments. Deep brain stimulation has shown promise as an intervention but has yielded inconsistent outcomes, potentially due to the complex biopsychosocial nature of MDD and the lack of standardized large animal models. The ventral striatum/ventral capsule brain area (VS/VC), involved in emotional and behavioral regulation, is a proposed DBS target. The Göttingen minipig (GM) is an emerging non-primate large animal model; however, the anatomy and connectivity of the GM VS/VC remain poorly characterized. To characterize the neuroanatomy and connectivity of the GM VS/VC region, we have used four female minipigs, which underwent MRI-guided stereotaxic unilateral injection of retrograde (FluoroGold) and anterograde (Biotinylated Dextran Amine) tracers into the VS/VC. Postmortem brains were coronally cryosectioned into 40 µm-thick sections, Nissl-stained, and analyzed for tracer distribution. Additional non-injected GM brains were immunostained using antibodies against calbindin, substance P, myelin basic protein, DARPP-32, tyrosine hydroxylase, and choline acetyltransferase to describe cytoarchitecture. The GM VS/VC exhibited bidirectional connectivity with limbic, associative, and sensorimotor regions, paralleling human nigrostriatal and mesolimbic circuits. The cytoarchitecture, particularly of the nucleus accumbens, is similar to that of primates, including the human brain. The GM VS/VC demonstrates anatomical and connectivity features similar to those found in humans and rodents, supporting its translational potential for modeling neuropsychiatric conditions and testing interventions such as DBS.

## Introduction

### Depressive disorder

The World Health Organization (World Mental Health Report 2022) estimates that 970 million people worldwide live with a mental disorder, with depressive disorders accounting for approximately 29% of these cases. Major depressive disorder (MDD) is a multifactorial, recurrent, and highly individual psychiatric condition, resulting from complex biopsychosocial interactions, with a lifetime prevalence of about 5 to 17% (Pedersen et al. 2014). It is associated with substantially reduced quality of life, increased morbidity, and premature mortality, with an average life expectancy reduction of roughly 7–10 years, and accounts for about 8–10% of all years lived with disability globally (Korhonen et al. 2021; Chan et al. 2023).

Although current treatment modalities, including psychotherapy, pharmacotherapy, and electroconvulsive therapy, are effective for many patients, a substantial proportion experience poor response. Among individuals with MDD who fail initial treatments, 30–50% do not benefit from alternative first-line antidepressants or combination therapies and are classified as having treatment-resistant depression (TRD) (Rush et al. 2006, 2020; Carvalho et al. 2014; Dodd et al. 2021). This underscores the urgent need for novel, effective interventions and the development of reliable translational models to study depressive disorders.

### Deep brain stimulation

Deep brain stimulation (DBS) is an established neurosurgical intervention for movement disorders such as Parkinson’s disease, essential tremor, and dystonia. More recently, DBS has been explored for psychiatric indications, including severe anxiety and obsessive-compulsive disorder. (Denys et al. 2020; Haber et al. 2021; Tyagi et al. 2022; Bandelow et al. 2023). In the context of TRD, chronic DBS has been investigated with electrode implantation in several deep brain regions, including the ventral striatum/ventral capsule (VS/VC) (Dougherty et al. 2015; Bergfeld et al. 2016; Lai et al. 2023), the subcallosal cingulate area (SCA) (Mayberg et al. 2005; Eitan et al. 2018; Merkl et al. 2018; Sankar et al. 2020; Mol et al. 2025), and other limbic structures (Drobisz and Damborská 2019; van der Wal et al. 2020; Blomstedt et al. 2017; Runia et al. 2025; Mol et al. 2025). However, randomized controlled trials have yet to demonstrate consistent clinical efficacy for TRD-DBS, likely due to unresolved questions regarding optimal anatomical targets, stimulation parameters, biomarker-guided localization, and the therapeutic effects of chronic versus adaptive (closed-loop) stimulation. Likewise, a case report (Scangos et al. 2021) demonstrated the potential of closed-loop neuromodulation in a patient with MDD, implanted into the VS/VC.

### VS/VC function

The ventral striatum comprises several interconnected structures, including the nucleus accumbens (Acb), a key limbic-motor interface of the basal ganglia (Lucas-Neto et al. 2013), along with the head of the caudate nucleus (NC), the anteroventral putamen (Pu), and the olfactory tubercle (Tu). The ventral capsule primarily encompasses the anterior limb of the internal capsule (ALIC). Dense dopaminergic connections from the substantia nigra pars compacta (SNc) and the ventral tegmental area (VTA) go through these regions, positioning the VS/VC as a critical hub in emotion regulation, motivation, and reward-guided behavior (Burton et al. 2015). Functional and neuroimaging studies support the involvement of the VS/VC in the pathophysiology of mood disorders (Quevedo et al. 2017). Positron emission tomography (PET) studies have reported reduced dopamine transporter availability (Meyer et al. 2001), decreased dopamine D1 receptor expression (Cannon et al. 2009), and increased striatal serotonin transporter binding potential (Cannon et al. 2007) in individuals with MDD compared to healthy controls. Given this evidence, the VS/VC remains a promising target for DBS in TRD, yet its translational modeling in large animals is underdeveloped. The VS/VC is also a target for neuromodulation in the treatment of obsessive-compulsive disorder (OCD), using DBS (Makris et al. 2016) or low-intensity transcranial focused ultrasound (Chou et al. 2025).

### The Göttingen minipig

The Göttingen minipig (GM) is an emerging non-primate large animal model in translational neuroscience (Sorensen et al. 2011). It offers several advantages, including a gyrencephalic brain, human-like cortical and subcortical cytoarchitecture (Bjarkam et al. 2017a), similar to the human white-to-gray matter ratio (Melia-Sorolla et al. 2020), compatibility with human-intended medical devices and imaging techniques, and lower ethical and financial burdens compared to non-human primates (Sorensen et al. 2011). Our research group has more than 25 years of experience using the GM model in studies of neuromodulation, neurodegenerative disease, and functional neuroanatomy, including investigations of DBS in Parkinson’s disease (Christensen et al. 2018). We have previously characterized the cytoarchitecture and retrograde connectivity of the GM Acb (Meidahl et al. 2016) and BA25 cortex homolog (Glud et al. 2024). However, the broader anatomy and connectivity of the GM VS/VC complex remain uncharacterized.

### Objective

This study aimed to provide a preliminary description of the neuroanatomy and connectivity of the GM VS/VC using MRI-guided stereotaxic neuronal tracing. These findings will provide the anatomical foundation necessary to support the development of a large animal model for DBS targeting the VS/VC in translational studies of TRD.

## Methods

### Animals

Four female GM (Ellegaard, Dalmose, DK), aged 8–9 months, and weighing 18.6–21.5 kg, were used for surgery as approved by the Danish National Council of Animal Research Ethics (protocol number 2021-15-0201–01078).

### Neuronal tracing

The tracing mixture contained 4% FluoroGold (FG, Hydroxystilbamidine bis(methanesulfonate), Sigma 39286-10MG-F) and 20% biotinylated dextran amine (BDA, NeuroTrace™ BDA-10.000 Neuronal Tracer Kit, Thermo-Fisher, USA), which served as the retro- and anterograde tracer, respectively. Both were dissolved in sterile distilled water in a 1:1 ratio to reach a mixture concentration of 2% FG and 10% BDA before injection.

All procedures were performed according to established protocols (Bjarkam et al. 2004; Ettrup et al. 2011). Briefly, the GMs were sedated using an intramuscular injection of S-ketamine (4 ml, 25 mg/ml) and midazolam (6 ml, 5 mg/ml). Intravenous access was gained through ear vein cannulation, permitting an additional infusion of the same sedative, after which the GMs were intubated and mechanically ventilated with 2–3% sevoflurane. The GMs were placed in a prone position, and the head was fixed in an MRI-compatible headframe (Mark 2.5, Neurologic, Denmark) with a TSE parallel rail and micromanipulator assembly (TSE System, GmbH, Bad Hamburg, Germany) and stabilized with bilateral zygoma screws. To ensure sufficient analgesia, the GMs received 2.5 ml Marcaine subcutaneously around each screw and 5 ml around the incision site, as well as 2 ml buprenorphine (Temgesic^®^) intramuscular. A midline incision was made to expose the skull, and a fiducial marker was inserted in a skull burr hole (Glud et al. 2017). The stereotaxic coordinates for each injection target were obtained individually for each animal with the fiducial as starting point on MRI using a Siemens Magneton Prisma Fit 3 T (Siemens, Germany) with 3D Turbo Flash T1 sequence as follow: voxel 1 × 1 × 1 mm, slice 176, FOV 256 × 256 pixels, TR: 2420ms, TE: 3.7ms, TI: 960ms, Flip: 9°, Averages: 2. Each animal received one, unilateral, right-sided tracer injection as follow: a craniotomy was performed using a neurosurgical drill (Midas, Medtronic, USA), and a 5 µL Hamilton syringe (Hamilton, USA) with an attached 22-gauge blunt cannula (outer diameter 0.72 mm), containing 1 µL of neuronal tracing mixture was placed in the micromanipulator assembly, before lowering the syringe to the target. 0.1 µL/min of tracer mixture was injected for 10 min, followed by a 3-minute resting period, 1 mm retraction of the cannula, a 1-minute resting period, and, finally, complete retraction to prevent reflux. When the injection was finished, the skin was closed with sutures.

### Perfusion and tissue preparation

After 28 days, the GMs were sedated as described above and euthanized by intracardial injection of pentobarbital overdose, followed by transcardial perfusion-fixation using 5 L of phosphate-buffered formaldehyde (PFA, 4%, pH 7.4) (Bjarkam et al. 2017b; Ettrup et al. 2011). The brains were carefully removed and immersed in the same fixative for at least 10 days. The fixated brains were then embedded in HistOmer^®^ alginate (Sorensen et al. 2000; Bjarkam et al. 2001), cut into 2 cm slabs, and cryoprotected for 8–10 days in a 30% sucrose solution. The slabs were frozen in isopentane cooled with dry ice to −50 °C, and then sectioned on a cryostat (CryoStar NX70, Thermo Scientific) into 40 μm-thick coronal sections. For every 20 sections, the first 2 series were mounted on gelatin-coated glass for Nissl staining and FG visualization, the next 3 series were kept free-floating in PBS for BDA visualization and future studies; the rest were discarded, yielding an inter-serial distance between the sections of 800 μm.

### Histology

FG did not require further processing. For an anatomical overview, FG sections were counterstained with toluidine blue in citrate buffer, dehydrated through 70%, 95%, and 99% ethanol, cleared in xylene, and coverslipped with Depex. To visualize BDA, free-floating sections were first incubated in 3% H_2_O_2_ with 0.1 M PB and 10% methanol to inactivate endogenous peroxidase activity. Sections were then preincubated in 0.2% milk in 0.1 M PB before incubation with the avidin-biotin complex solution (ABC, VECTASTAIN ^®^ Elite ABC kit, Vector Laboratories, USA). BDA labeling was visualized using a DAB solution (Kem-En-Tec Nordic A/S, Taastrup, DK), and sections were mounted on gelatin-coated glass slides.

Brain tissue from additional GMs that did not receive tracer injections was processed for immunohistochemistry using antibodies against tyrosine hydroxylase (TH), dopamine- and cAMP-regulated phosphoprotein 32 kDa (DARPP-32), choline acetyltransferase (ChAT), myelin basic protein (MBP), substance P (SP), and calbindin D28k (CB) (Table 1).

Briefly, free-floating sections were rinsed with Tris-buffered saline (TBS, 0.05 M, pH 7.4) with 1% Triton X-100 (TBS-T), followed by target retrieval in DAKO buffer (DAKO S1699) at 80 °C water bath for 30 min. Endogenous peroxidase activity was quenched using a peroxidase-blocking solution. Sections were then preincubated with either 0.1% avidin + 0.01% biotin, 0.2% milk, or 1% horse serum to block nonspecific binding, followed by incubation with primary antibody diluted in TBS-T containing either 0.02% milk or 1% horse serum. One section per series was processed without a primary antibody as a negative control. Sections were incubated overnight at 4 °C. The following day, secondary antibodies were applied. The ChAT, CB, MBP, and SP-labeled sections were incubated in ABC solution for 1 h at room temperature. DARPP-32-labeled sections were incubated in avidin-peroxidase solution. All sections were visualized using diaminobenzidine (Kem-En-Tec Nordic A/S, Taastrup, DK) before mounting on gelatin-coated glass slides.

Anatomical descriptions and structure identifications were done by the same investigator, using a Leica DM5000B microscope with attached DFC480 camera, based on the online GM Brain Atlas (Orlowski et al. 2019), published at cense.au.dk, and existing literature (Meidahl et al. 2016; Bjarkam et al. 2017a; Steinmuller et al. 2021; Glud et al. 2024; Felix et al. 1999). Likewise, the anatomical terminology and abbreviations used here follow previous studies on minipig brain anatomy (Bjarkam et al. 2017a; Orlowski et al. 2019). The FG tracing intensity presented in Table 2. was arbitrary categorized according to the amount of visible FG-positive cells in the target areas into three categories: + (weak) - few cells visible (as in the Fig. 2a, d,g, j); ++ (moderate) - moderate number of cells visible in the field of view (Fig. 2b, e,h, k); +++ (strong) - many cells visible in the single field of view (Fig. 2c, f,i, l); with an additional fourth category, ++++, for the injection areas. BDA tracing (Table 3), due to the relatively low number of axons visible in the target areas, was recorded as positive or negative. As the ALIC is a white-matter region, the observed tracer signal was interpreted as originating from the interwoven gray matter adjacent to the ALIC, with possible uptake also by the fibers-of-passage.

**Table 1:** Antibodies in this study

Primary antibody	Supplier	Animal source	Dilution	Secondary antibody	Dilution	Visualization protocol
CB	Abcam (ab82812)	Mouse, mAb	1:1000	Goat-anti-mouse, biotinylated, Abcam (ab97033)	1:200	ABC/DAB
TH	Abcam (ab112)	Rabbit, pAb	1:1000	Goat-anti-rabbit, HRP, DAKO	1:400	DAB
DARPP-32	Abcam (ab40801)	Rabbit, pAb	1:15000	Goat-anti-rabbit, biotinylated, Abcam (ab6720)	1:400	DAB
SP	Enzo (BML-SA1270)	Rabbit, pAb	1:1000	Goat-anti-rabbit, biotinylated, Abcam (ab6720)	1:200	ABC/DAB
ChAT	Millipore (AB144P)	Goat, pAb	1:2000	Horse-anti-goat, biotinylated, Vector Labs (BA-9500)	1:200	ABC/DAB
MBP	Biolegend (SMI 94)	Mouse, mAb	1:200	Goat-anti-mouse, biotinylated, Abcam (ab97033)	1:200	ABC/DAB

*CB:* Calbindin D-28k; *TH:* Tyrosine hydroxylase; *DARPP-32:* Dopamine and cAMP-regulated phosphoprotein 32 kDa; *SP:* Substance P; *ChAT:* Choline acetyltransferase; *MBP:* Myelin basic protein; *mAb:* Monoclonal antibody; *pAb:* Polyclonal antibody; *DAB, 3,*3’-Diaminobenzidine tetrahydrochloride; *ABC:* Avidin-Biotin Complex Vectastain kit; *HRP:* horseradish peroxidase

## Results

### Neural connectivity (Tables 2 and 3)

**Fig. 1:**
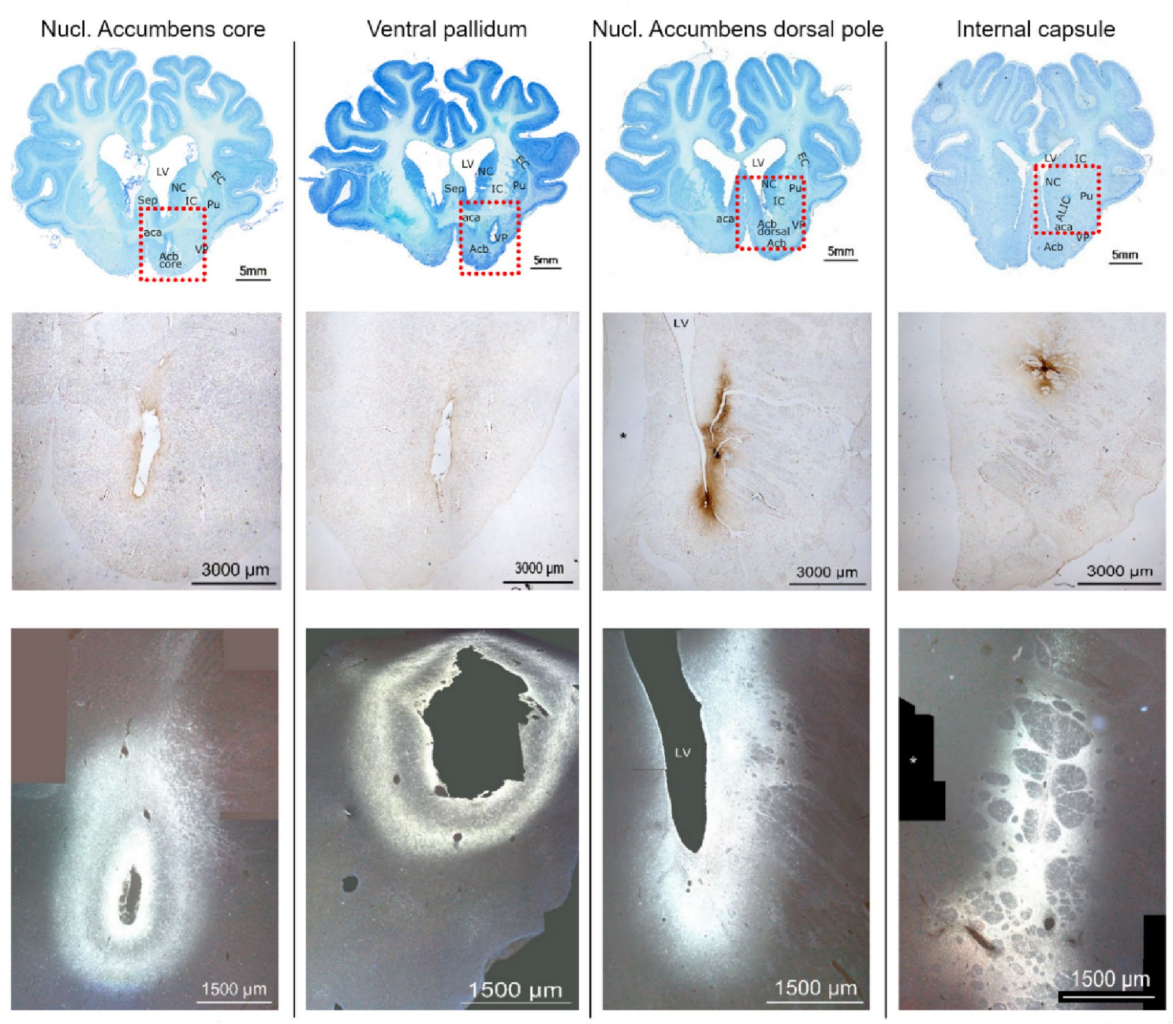
Injection sites. Upper row: Nissl stain, injected areas marked with a red box; middle row: BDA visualization of the injected areas; lower row: FG visualization. Signs of inflammation and surgical insult can be seen as missing tissue with surrounding macrophages and hemosiderin sediments. BDA 1.5x objective magnification; FG 5x objective magnification. *Missing fragment of microphotograph. LV, lateral ventricle

The injection targets were placed in the four animal brains (named AVS1-4) as follows: in the Acb core, in ventral pallidum (VP), in Acb dorsal pole (dorsal part of the Acb, above the core), and ALIC, as shown in Fig. 1. Microscopic examination of tissue along the injection tracts above the injection target, revealed low uptake of BDA by neuronal cell somas in brains AVS1 and AVS4. In brain AVS2, limited uptake was noticeable in the striatum/internal capsule area, whereas in brain AVS3, additional uptake was visible mainly along the IC, right above the injection site.

#### Retrograde connectivity

**Fig. 2:**
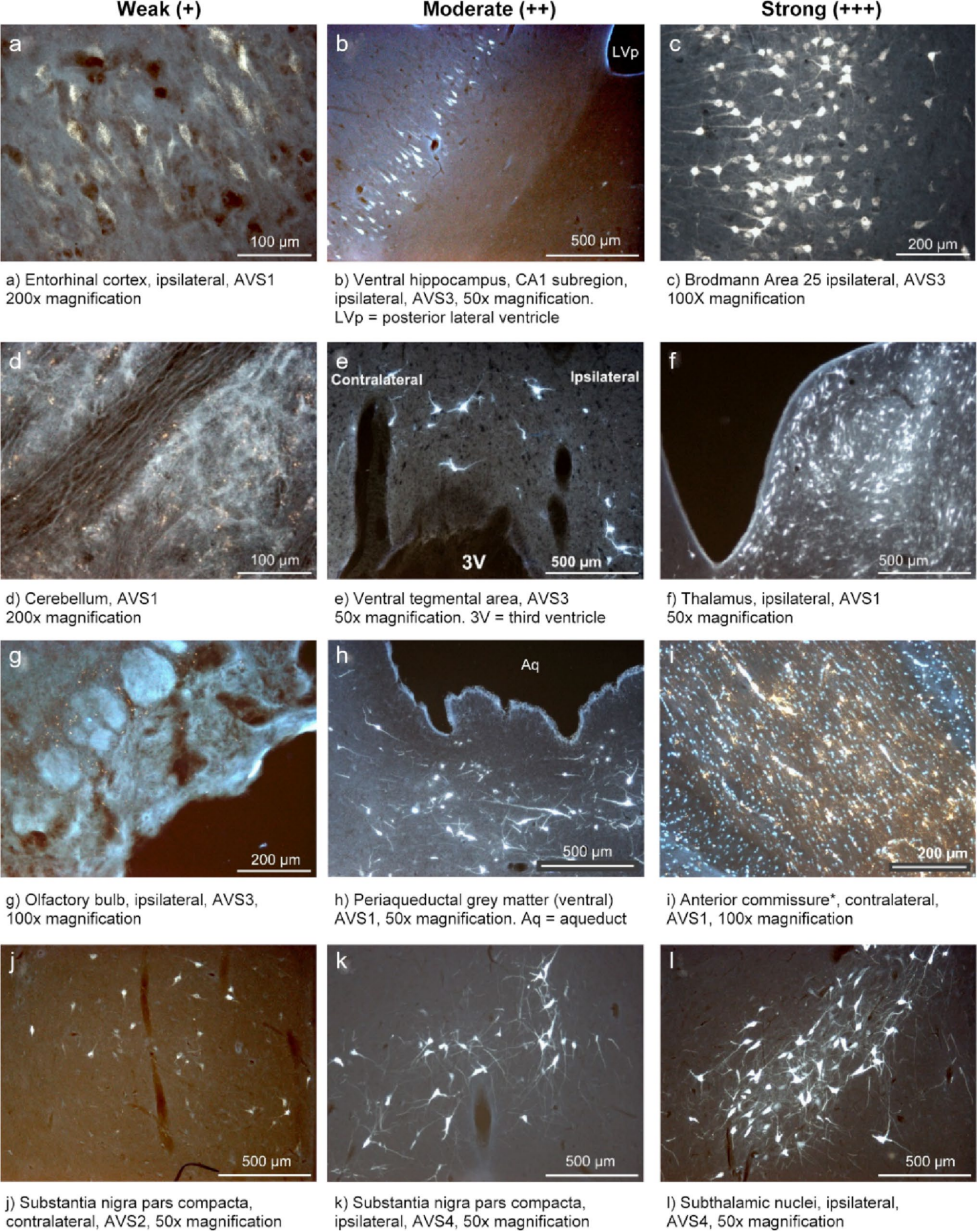
Examples of the intensity of FG tracer projections. Each column shows tracing intensity in different areas: weak, moderate, and strong, respectively. *In white matter structures, only the passing fibers can be seen; AVS1-4 denote the animal.

Our retrograde tracing revealed a network of projections to the GM VS/VC, with ipsi- and contralateral labeling observed across several key brain regions (Table 2.; Fig. 2).

All injection sites showed both ipsi- and contralateral retrograde projections originating from the mesencephalon, including specific input from the periaqueductal gray (PAG), particularly to the nucleus accumbens core and dorsal pole. Additional retrograde labeling was consistently observed in the thalamus (Thal), SNc, VTA, SCA, visual cortex, and medial agranular prefrontal cortex (mPFC). FG-positive fibers were also seen traversing major white matter tracts, including the anterior commissure (aca), corpus callosum, and telencephalic white matter (TWM).

The VP area received strong ipsilateral retrograde projections via the internal capsule (IC) from the amygdala (Amyg), Acb, NC, Tu, and the anterior medial olfactory nucleus. Additional inputs arose from the anteromedial and central medial thalamic nuclei. Projections to the VP originated from the ipsi- and contralateral globus pallidus (GP), Thal, entorhinal cortex, and temporal cortex.

Retrograde labeling in the anterior limb of the internal capsule was primarily ipsilateral, including inputs from the septum (Sep), SCA, Thal, and mPFC, with fibers traveling through the aca and external capsule (EC). Retrograde projections to the ALIC from both hemispheres originated in the visual cortex and SNc; notably, the ALIC was the only site to exhibit bilateral projections from the subthalamic nucleus (STN).

A comparison of FG labeling in the Acb core and dorsal pole revealed overlapping but distinct connectivity patterns. Motor cortex and NC projections were exclusive to the dorsal pole, whereas inputs from the Pu, VP, Tu, horizontal and vertical limbs of the diagonal band (HLDB and VLDB), and cerebellum were only observed in the core. While both subregions received ipsilateral thalamic projections, the specific thalamic nuclei involved differed by injection site. Sparse retrograde labeling also originated from the ipsilateral claustrum.

Both Acb subregions shared input from the Amyg, GP, ventral hippocampus (vHip), amygdalohippocampal transition area, and Sep. Cortical inputs to both sites included the dorsal and ventral somatosensory, entorhinal, temporal, granular dorsal, and agranular ventral insular cortices. Projections from various olfactory areas in both hemispheres were observed, except for the Tu, which projected only to the core.

#### Anterograde connectivity

**Fig. 3:**
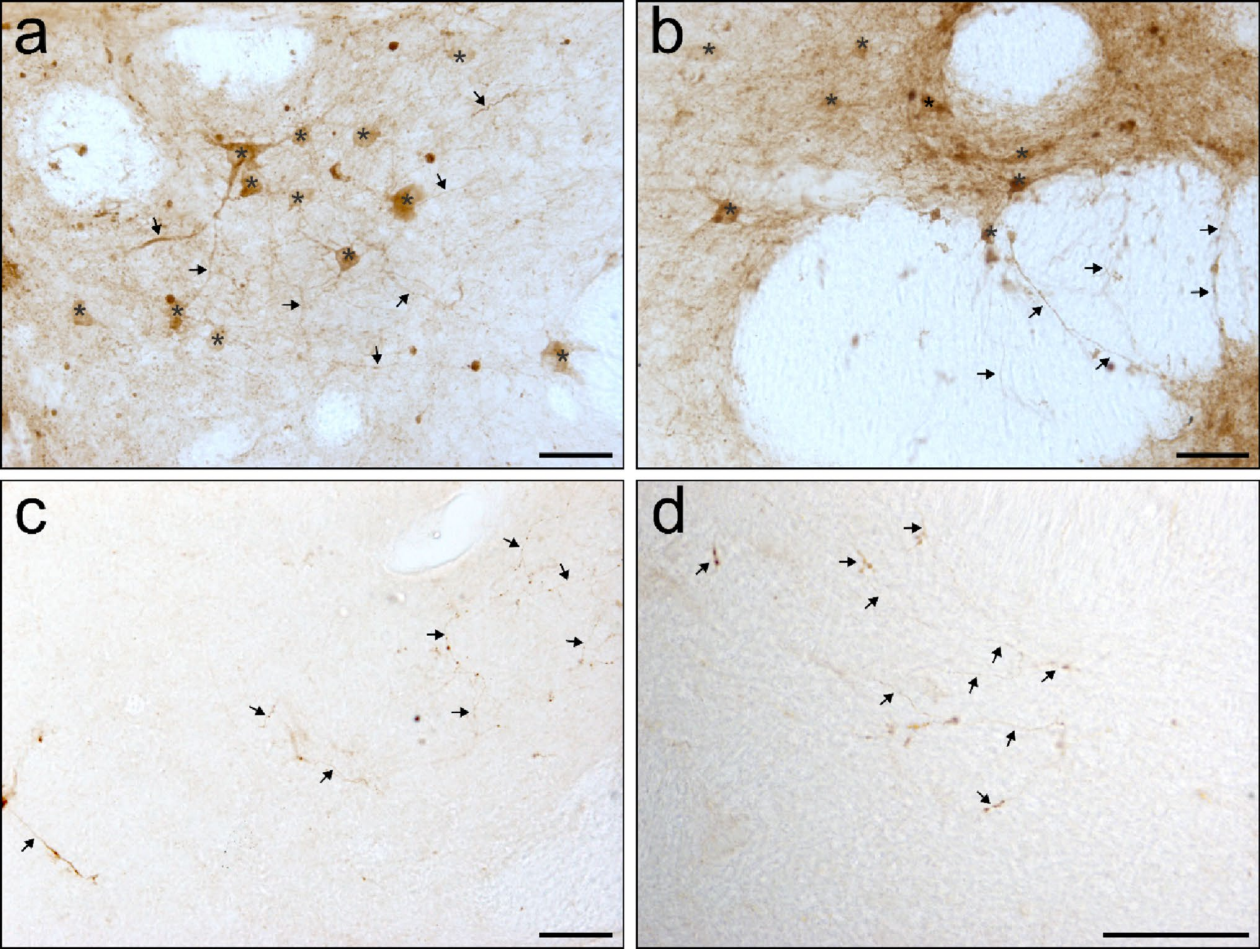
Examples of the BDA tracing. **a** AVS3, injection area (dorsal Acb); **b** AVS4 injection area (ALIC); **c** AVS4, tracing to caudate nucleus; **d** AVS4 tracing to thalamus; stars * indicate cell bodies, which uptake BDA, arrows indicate BDA-positive axons, scale bars = 50µm

Anterograde tracing with BDA showed a more limited distribution (Table 3), with only a small number of labeled axons observed in the target areas (Fig. 3c, d). Moreover, the number of cells that uptake the BDA in the injection sites was also limited (Fig. 3a, b). Likewise, the BDA staining intensity outside areas proximal to the injection sites was low, which made analysis of the anterograde labeling difficult (Fig. 3). Nevertheless, all injection sites demonstrated anterograde projections to the ipsilateral NC. Projections from both the Acb core and dorsal pole passed through the TWM and IC, with terminal fields identified in the ipsilateral Tu, Pu, and Thal. Additionally, fibers from the Acb core were observed traversing the EC, whereas projections via the hippocampal commissure were unique to the dorsal pole.

The VP injection resulted in BDA-labeled fibers in the ipsilateral prepiriform cortex (PrPir), nucleus of the lateral olfactory tract, EC, IC, Acb, Tu, and Pu, as well as in the contralateral temporal cortex.

**Fig. 4:**
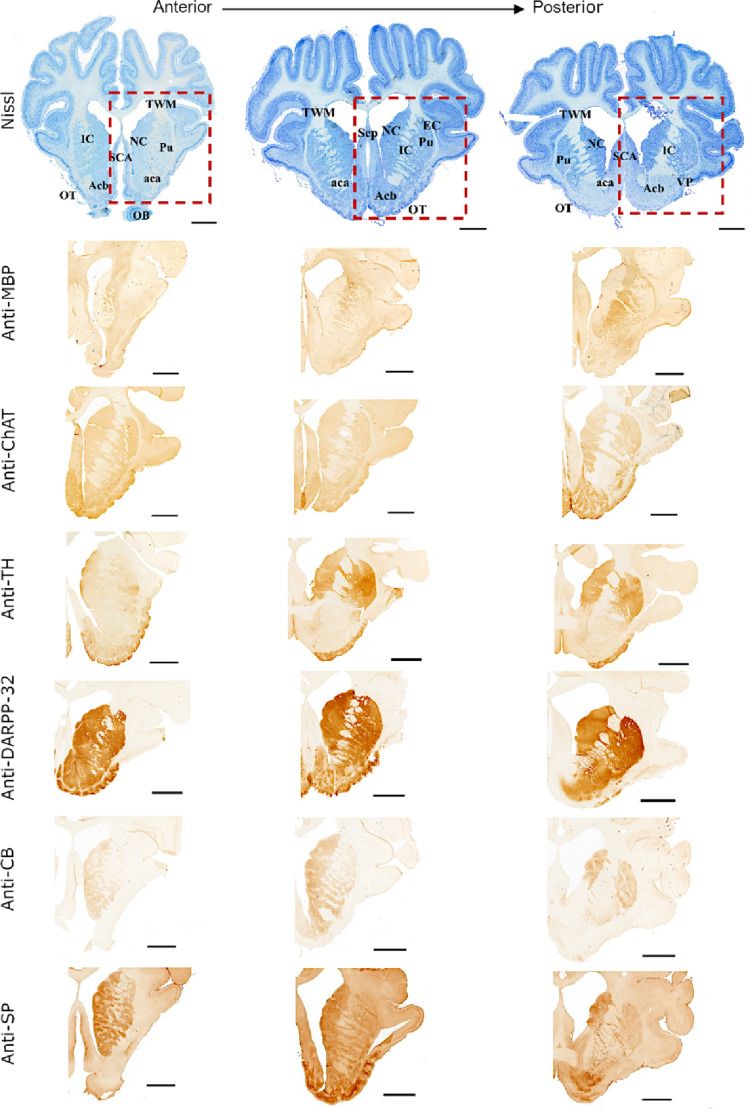
Immunohistochemistry: representative examples of the VS/VC area labeled with Nissl stain and various antibodies. Coronal sections are presented in three different sagittal planes. The presented immunostained sections do not represent the same plane as the Nissl stains. Scale bar = 5 mm

Finally, BDA injections into the ALIC produced anterograde labeling in the ipsilateral Acb and Thal (Fig. 4d).

For a comprehensive overview of retrograde and anterograde connections, see Tables 2. and 3, and representative micrographs in Fig. 2, showing variable FG intensity, and Fig. 3 with examples of the BDA tracing.

**Table 2:** FluoroGold tracing from each injection target. Sorted alphabetically

	Nucleus accumbens core (AVS1)		Ventral pallidum (AVS2)		Nucleus accumbens dorsal pole (AVS3)		Internal capsule ventral, ALIC* (AVS4)	
	Ipsilat.	Contralat.	Ipsilat.	Contralat.	Ipsilat.	Contralat.	Ipsilat.	Contralat.
Amygdala	++	+	++		++			
Anterior commissure (Anterior crus)*	+++	+++	+++	+	+	+	+	
Caudate nucleus, head			++		++			
Central telencephalic white matter*	+	+	+	+	+	+	+	+
Cerebellum	+m	+m						
Claustrum	+				++			
Corpus callosum*	+m	+m	+m	+m	+m	+m	+m	+m
Cortex, entorhinal	+	+	++	+	+			
Cortex, insular (agranular ventral)	+				+			
Cortex, insular (granular dorsal)	+				++			
Cortex, motor					+	+		
Cortex, prefrontal (medial agranular)	+		++		+++		++	
Cortex, somatosensory (dorsal)	+	+			+	+		
Cortex, somatosensory (ventral)	+	+			+	+		
Cortex, temporal		+	+	+	+	+		
Cortex, visual		+	+	+	+	+	+	+
Diagonal band, horizontal limb	++	+	++					
Diagonal band, vertical limb	+++	+						
External capsule*	+	+			++	+	+	
Globus Pallidus	++		++	+	+			
Hippocampus, ventral CA1 subregion	++				++			
Internal capsule*	++	++	++		+	+	++++i	+
Mesencephalon	++	+	++	+			++	+
Mesencephalon, periaqueductal grey matter	++	+			++	++		
Nucleus Accumbens	++++i	++	+		++++i			
Olfactory bulb	+	+			+	+		
Olfactory bulb, accessory	++	+			+	+		
Olfactory nucleus, anterior	++	++			++	+		
Olfactory nucleus, anterior medial part	++	+++	+		+	+		
Olfactory nucleus, anterior lateral part	++	++			++	+		
Olfactory nucleus, anterior posterior part	++				+			
Olfactory tubercle	++	+	+					
Putamen	+	+						
Septum	+				+		+	
Subcallosal cingulate area, BA25	+++		++		+++	+	+	
Substantia Nigra, pars compacta	++	+	++	+	++	+	++	+
Subthalamic nuclei							+++	++
Thalamic nucleus, anterodorsal	++				+	+		
Thalamic nucleus, anteromedial			+		+++			
Thalamic nucleus, anteroventral	++							
Thalamus	+++		+	+	+		++	
Ventral pallidum	++		++++i					
Ventral tegmental area	+++m	+++m	+m	+m	++m	++m	+m	+m

Projections are categorized as either ipsilateral, contralateral, or bilateral. Structures in which the hemispheres could not be segregated are considered midline, shown by ‘+m’ in both the ipsilateral and contralateral columns. Projection intensity is graded as weak (+), moderate (++), or strong (+++) compared to injection site intensity (++++i); examples are shown in Fig. 2. *Positive tracing in white matter areas should be interpreted as fibers passing through the structure, thus not as a target structure

**Table 3:** BDA projections from each injection target

	Nucleus accumbens core (AVS1)		Ventral pallidum (AVS2)		Nucleus accumbens dorsal pole (AVS3)		Internal capsule ventral, ALIC (AVS4)	
	Ipsilat.	Contralat.	Ipsilat.	Contralat.	Ipsilat.	Contralat.	Ipsilat.	Contralat.
Caudate nucleus, head	X		X		X		X	
Central telencephalic white matter*	X				X			
Cortex, prepiriform			X					
Cortex, temporal				X				
External capsule*	X		X					
Hippocampal commissure*					Xm	Xm		
Internal capsule*	X		X		X		Xi	
Mesencephalon	X							
Nucleus Accumbens	Xi	X	X		Xi	X	X	
Nucleus of the Lateral Olfactory Tract			X					
Olfactory tubercle	X		X					
Putamen	X		X					
Thalamus	X						X	
Ventral pallidum			Xi					

Injection sites are marked as Xi. Positive anterograde tracing from the injected areas is marked with X. Tracing intensity was not assessed in BDA sections due to sparser distribution and weaker visualization; in the table presented are areas where tracing was observed. *Positive tracing in white matter areas should be interpreted as passing through the structure, thus not as a target itself. Structures in which the hemispheres could not be segregated are considered midline, shown by ‘Xm’ in both the ipsilateral and contralateral columns

### Neuroanatomy

A summary of the immunohistochemical results is provided in Fig. 4.

#### *Caudate nucleus*,* putamen*,* and internal capsule*

As shown in Fig. 4, the head of the caudate nucleus and the putamen were identifiable as distinct, DARPP-32-immunoreactive structures, located bilaterally on either side of the internal capsule. Anteriorly, both nuclei are elongated and narrow, gradually widening and losing their longitudinal orientation in posterior sections.

Both the NC and Pu are immunoreactive for substance P and calbindin, most prominent in the NC and the posterior Pu. TH-positive fibers and synaptic clusters were distributed throughout both nuclei. In ChAT-labeled sections, scattered cholinergic neurons were observed within the Pu.

The anterior limb of the internal capsule was consistently identified in all animals, extending ventrally from the central telencephalic white matter as an MBP-positive, cell-sparse tract interspersed with MBP-negative cell-dense striae.

#### *Nucleus accumbens*

Acb, as described previously (Meidahl et al. 2016), appeared in anterior coronal sections as a large, rounded structure located ventral to the IC. In more posterior sections, it became separated from the IC by the anterior commissure and appeared shifted to a more ventral position. The shell region could be delineated medially, ventrally, and laterally from surrounding structures. However, its borders were not sharply demarcated. Rostrally, the dorsal pole of the Acb merged with the NC and Pu, without a clear anatomical boundary. Likewise, precise delineation of the border between the Acb core and shell was not possible using TH-, DARPP-32-, and ChAT-labeled sections. CB immunoreactivity was stronger in the core and gradually faded toward the CB-poor shell, resulting in a gradual transition rather than a distinct boundary between these subregions. SP-positive neurons were distributed throughout the Acb, without consistent differences between the presumed core and shell regions.

#### *Subcallosal cingulate area*,* septum*,* and medial septal complex*

SCA and Sep were readily identifiable; the SCA contained ChAT-positive neurons and displayed clear borders separating it from the adjacent MBP-positive and ChAT-positive medial septal complex (MSC). No DARPP-32 or TH immunoreactivity was detected in the SCA, Sep, or MSC, suggesting limited or absent dopaminergic input to these regions in the Göttingen minipig. Scattered CB- and SP-positive neurons were observed throughout the septal complex.

#### *Ventral pallidum*

VP, present in posterior sections, was not clearly demarcated, emerging ventrally to the posterior crus of the anterior commissure. It is located dorsolateral to the Acb as an oval, diagonal nucleus, perpendicular to the olfactory tubercle (Tu). The VP exhibited positive DARPP-32, and TH staining alongside scattered, sparse ChAT-positive cell bodies and some ChAT-positive fibers, but lacked immunoreactivity for MBP, CB, and SP.

#### *Olfactory tubercle*

The characteristic convoluted surface of the Tu was well visualized in Nissl and anti-TH, -DARPP-32, and -SP labeled sections, with abundant pre- and postsynaptic dopaminergic elements present throughout the profound layers. Mild anti-ChAT positivity was noted in the superficial layer. The characteristic islands of Calleja were readily visible in Nissl stain and ChAT-, TH-, DARPP-32-, and SP-labeled sections. Notably, no MBP or CB immunoreactivity was observed in the Tu, which distinguishes it from the adjacent ventral shell of the Acb and the VP.

#### *Other olfactory structures*

Olfactory structures were mostly absent from the immunohistochemically processed material. In a few anterior sections,

the olfactory bulb (OB) was partially visible and displayed moderate anti-MBP and anti-SP immunoreactivity at its external boundaries. Nissl staining revealed a characteristic trilaminar organization of the OB. The MBP-positive lateral olfactory tract and subsequently the MBP-poor PrPir were both ChAT-, DARPP-32-, and TH-negative. While the LO did not show anti-SP labeling, the laterally adjacent PrPir exhibited scattered SP-positive neurons. Small clusters of CB-positive cells were observed in the posterior part of the anterior olfactory nucleus in most anteriorly analyzed sections.

#### *Anterior commissure*

The anterior commissure (aca) was readily visualized across all used staining modalities. In the sagittal plane, it traverses from a ventrolateral to dorsomedial trajectory. The posterior limb of the anterior commissure appeared as an MBP-positive and was negative for CB-, SP-, and TH staining.

## Discussion

### Neural connectivity

This study, summarized in Tables 2. and 3, aimed to characterize the anatomy and connectivity of the VS/VC region in the GM, using a combination of tract-tracing and immunohistochemical techniques. Our results indicate that the minipig VS/VC is receiving connections from the limbic, associative, and sensorimotor regions, which may be consistent with a role in affective and behavioral regulation. Our limited anterograde data suggest that at least some of those connections could be bidirectional. The presented findings highlight the topographically organized connectivity of this region, involving cortical, subcortical, and brainstem structures. Together, these observations provide anatomical insights that may be relevant for translational research, including studies of neuromodulatory interventions such as deep brain stimulation. In the following sections, we focus on the central pathways most relevant to MDD. It is important to note that the VS/VC region contains major white matter tracts, including the anterior limb of the internal capsule and the medial forebrain bundle. Consequently, tracer labeling in this region may reflect uptake from both interwoven gray matter and fibers of passage. This represents a limitation for interpreting direct synaptic connectivity. However, it does not preclude the functional relevance of this region, particularly in the context of network-level interactions. From a clinical neuromodulation perspective, targeting regions with traversing fibers can still yield therapeutic effects by modulating distant but functionally connected brain structures (Makris et al. 2016).

#### *Basal ganglia loops*

The dopamine transmission is essential for the function of the basal ganglia circuits, with SNc serving as a major output nucleus to D1 and D2 neurons of the dorsal striatum in humans and other mammals through the nigrostriatal pathway (Hedreen and DeLong 1991; Feuerstein et al. 1989; Gagnon et al. 2017; Sharples et al. 2020). Disturbances in this circuit are often linked to movement disorders, but the complex interactions between the basal ganglia and limbic circuits, involving both the SNc and the VTA, indicate that the nigrostriatal pathway may also be involved in neuropsychiatric pathology (McCutcheon et al. 2019). Interestingly, observed by us, ipsi- and contralateral projections from the SNc to the VS/VC in the GM suggest a pathway akin to the nigrostriatal pathway, although the limited BDA distribution prevented thorough anterograde investigation in this study. Those results, together with positive TH staining for dopaminergic fibers in VS, confirm our previous studies on dopaminergic connections in GM, using anti-TH staining (Christensen et al. 2018).

Previous studies have shown that the Acb exhibits regional differences in neural connectivity. In both primates and rodents, direct pathway neurons originating from the ventromedial part of the Acb’s dorsal pole project to the ventromedial SNr, whereas neurons from the Acb core project predominantly to the VTA (Brog et al. 1993; Salgado and Kaplitt 2015; Castro and Bruchas 2019). These findings support the existence of functionally distinct, yet interconnected, subregions within the Acb. In the present study, due to the limited distribution of BDA, we did not observe anterograde projections to the VTA or SNr. However, we observed indications of interregional differences within the GM Acb: the presumed Acb core projected to the Thal, mesencephalon, Pu, and olfactory tubercle, while such projections were not observed from the dorsal pole of the Acb. These differences were also evident in the retrograde tracing results, although both the core and dorsal part of the Acb regions received overlapping afferents from several brain structures. These observations should be interpreted cautiously, given the methodological limitations. The human Acb is known to receive input from the entorhinal, motor, and sensory cortices, as well as the Thal, vHip, and BA25 (Salgado and Kaplitt 2015), connections that were all, to some degree, displayed in our current findings, as well as in previous studies (Meidahl et al. 2016). However, some of those connections may require further, more refined studies to confirm, since our other previous research, concerning the subcallosal cingulate area (BA25 homolog) of the GM cortex, did not show connections to Acb (Glud et al. 2024), possibly because the tracing of the small axons using anterograde BDA labeling in light microscopy is not always precise enough.

A previous minipig tracing study demonstrated efferent connectivity from the dorsal PFC and motor cortices to the ipsilateral dorsal striatum and the dorsolateral pole of the Acb in a similar pattern to primates (Steinmuller et al. 2021). In the present study, we found connections originating in the mPFC and visual cortex to all injected areas, while the insular and somatosensory cortices projected to the Acb, and the motor cortex projected only to the Acb dorsal part, thus adding to previous findings. Our retrograde findings in those areas suggest that the varying functional and connective properties of the Acb subregions may exist in the GM as well, which was also suggested before (Meidahl et al. 2016). Additionally, observed by us projections from the mPFC, GP, and Amyg to both GM Acb areas are similar to those seen in the rat brain (Baik 2020).

Finally, in the previous studies on the human brain, the ALIC has been shown, using parcellations and tractography, to be traversed by organized tracts from the prefrontal cortex (Nanda et al. 2017), Thal, and STN (Banks et al. 2022), as well as the Acb, hippocampus (Hip), and Amyg (Coenen et al. 2020). Although we did not observe anterograde labeling to the amygdala or hippocampus, we did identify bidirectional connections between the VS/VC and the ipsilateral thalamus, as well as retrograde projections from the mPFC, and from the STN via fibers traversing the ALIC. These observations are broadly consistent with previous studies reporting bidirectional connectivity through the ALIC in the GM. Taken together, our findings indicate that basal ganglia connectivity in the GM is similar to that described in non-human primates and humans. This supports the potential relevance of the GM for translational neuroscience research, while acknowledging that further work is needed to confirm functional equivalence.

#### *The mesolimbic pathway*

The dopaminergic outputs within the mesolimbic pathway, originating in the VTA, are implicated in a range of functions, including motivational behavior, stress coping, modulating perceived salience, and reward-prediction error. These roles have been demonstrated across species, including rodents, non-human primates, and humans (Baik 2020; Derdeyn et al. 2021; Carruzzo et al. 2023). In our study, retrograde VTA projections were observed at all injected sites, displaying a pattern similar to the known mesolimbic pathway of humans and rats. Beyond the VTA - Acb connectivity, the observed projections to the VP are also of particular relevance. As a central component of the mesocorticolimbic system, the VP is believed to modulate the mesolimbic pathway and has been shown to play a crucial role in reward-seeking behavior and addiction mechanisms in rats (Mahler et al. 2014). In non-human primates (macaque monkey), the VP has also been implicated in linking expected reward value to the execution of goal-directed action (Tachibana and Hikosaka 2012). In our material, we identified retrograde projections from the VTA to the injection site in VP. Anterograde connections from VP to VTA, although reported in other species (Mahler et al. 2014), were not confirmed in our study, possibly due to limitations in BDA tracer distribution; however, species-specific differences cannot be excluded. As previously mentioned, anterograde BDA tracing, especially to distant targets in a relatively large minipig brain, is more susceptible to technical limitations and may not reliably reflect the full extent of projections. Further studies are needed to clarify whether these absent projections in the GM reflect biological differences or methodological constraints. Nevertheless, some evidence from our study supports the presence of bidirectional connectivity between the Acb and VP in the GM, consistent with findings reported in the macaques (Hedreen and DeLong 1991) and rodents (Pardo-Garcia et al. 2019; Smedley et al. 2019). These structures are central to integrating motivational and reward-related signals and translating them into motor output (Smith et al. 2009). While our tracing did not reveal projections from the VP to the motor cortex, we did identify retrograde inputs from the entorhinal, prefrontal, and visual cortices to the VP, along with anterograde projections to the PrPir and bidirectional connections with the temporal cortex (contralateral temporal cortex connections with VP confirmed by both FG and BDA tracing).

#### *Hippocampus and amygdala*

As part of the limbic structures, the functionally heterogeneous hippocampus is involved in a range of complex cognitive and emotional regulatory processes. The vHip, in particular, has been implicated in context coding, stress-response, addiction-related behavior, and depressive pathology (Gulyaeva 2019; Bakoyiannis et al. 2023; Turner et al. 2022). In rats, studies have shown that the small pyramidal neurons in the vHip CA1 subregion are activated in anxiogenic environments requiring avoidance behavior (Gulyaeva 2019) and that the glutamatergic projections from the vHip to the Acb play a crucial role in driving chronic social defeat stress and depressive-like behavior (Bagot et al. 2015). In our study, we observed retrograde projections specifically from the ipsilateral vHip CA1 subregion to the Acb, suggesting that this region may play a functionally similar role in the GM as in rodents. The intricated connectivity between the basal ganglia and the limbic system has long been recognized. Notably, previous reports showed that the striatum, in particular the Acb and ventral parts of NC, receives input from several limbic structures, including vHip and Amyg, and projects to the SNc, thus forming a key motor-limbic interface (Buot and Yelnik 2012; Morrison et al. 2017). Retrograde projections from the amygdala to the Acb and VP are thought to modulate emotional and motivational processes (Soares-Cunha and Heinsbroek 2023). In our study, we found retrograde projections primarily from the ventral parts of the Amyg and the amygdalohippocampal transition area to the GM Acb and VP. Alongside the previously mentioned SNc connections, these findings support the existence of a conserved circuit architecture in the GM comparable to that observed in rodents and non-human primates. Our results are broadly consistent with previous investigations into the GM limbic system using MRI tractography (Bech et al. 2020), and add further support to the potential translational relevance of this model in neuropsychiatric research.

#### *Periaqueductal grey matter*

We observed FG labeling in the dorsal and dorsolateral parts of the PAG projecting to the core and dorsal part of the Acb. The PAG is a mesencephalic structure involved in regulating homeostatic neurophysiological functions, such as pain modulation, cardiorespiratory control, and anxiety-related responses (Behbehani 1995; Reis et al. 2023). Notably, the dorsolateral and dorsomedial regions of the PAG have been implicated in mediating panic-like behavior in rodents and panic attacks in humans (Quintino-dos-Santos et al. 2014; Falconi-Sobrinho et al. 2024). Although the functional organization of the PAG varies across species, a study in humans reported striatal connections specifically originating from the dorsolateral PAG (Coulombe et al. 2016). This is consistent with our findings in the GMs, which revealed retrograde projections from the PAG to the Acb, suggesting a comparable pathway that may be relevant for translational research.

#### *Cortical areas and the cerebellum*

Although relatively sparse, we detected some retrograde labeling from the cerebellum to the Acb core in animal AVS1. Interestingly, a recent study examining human cortico-striatal functional connectivity demonstrated that the ventral striatum is heavily engaged in reward anticipation, facilitated by its interactions with several cortical areas, including the cerebellum, motor, and visual cortex, which are believed to support motor responses to rewarding stimuli (Carruzzo et al. 2023). Our preliminary observation of cerebellar input to the Acb in GMs may suggest a comparable integrative role in sensorimotor and reward-related processes; however, this finding requires confirmation in further, more detailed studies. Although the functional relevance of these projections requires further investigation, recent evidence suggests that the cerebellum regulates emotion through wide cortical and subcortical networks, just as it contributes to emotional learning, especially in fear conditioning. Cerebellar dysfunction can cause emotional blunting and is linked to disorders like autism and ADHD (Rudolph et al. 2023).

In summary, the retrograde and anterograde projections identified in the GM VS/VC in this study suggest that this region participates in a complex set of neural circuits. The observed connectivity patterns appear broadly consistent with those described in humans, non-human primates, and rodents, particularly with respect to cortical, limbic, and associative networks. Taken together, these findings may indicate that the GM may represent a useful large animal model for investigating the neuroanatomical substrates relevant to psychiatric and neurological interventions, including deep-brain stimulation.

### Neuroanatomy

The VS/VC has been extensively described across species, including birds, reptiles, rodents, non-human primates, and humans (Medina and Reiner 1995; Park et al. 2019; Makris et al. 2016). In the GM, the VS/VC displays a topographical organization that shows partial similarity to that of the human brain, particularly regarding the anatomical arrangement of the Acb, caudate nucleus, putamen, and IC (Holt et al. 1997). The Acb in GMs appears to show a discernible demarcation at its medial, ventral, and lateral borders, allowing it to be distinguished from surrounding regions such as the Tu, Sep, and SCA, and in more posterior coronal sections, the VP. However, in our staining, the VP does not exhibit well-defined borders, and its delineation from adjacent structures should therefore be interpreted with caution. Unlike in humans, where the VP is located dorsally to the Acb near the bed nucleus of the stria terminalis, in GM it is positioned more laterally to the Acb and dorsally to the Tu. Contrarily, the rodent VS is structurally less differentiated, with the caudate and putamen nuclei merged into the caudate-putamen complex, positioned dorsally to the Acb, and the bed nucleus of the stria terminalis occupying a relatively larger portion of the region (Paxinos 2015).

Our group has previously described the neurochemical anatomy of the GM Acb using anti-DARPP-32 and anti-CB immunohistochemistry (Meidahl et al. 2016). The current study expands on that work by including additional markers: anti-TH, -SP, -ChAT, and -MBP, to further characterize the VS/VC region.

DARPP-32, a phosphoprotein expressed in dopaminoceptive neurons, is particularly abundant in striatopallidal and nigrostriatal projection neurons and plays a key role in modulating cellular response to dopaminergic signaling via inhibition of protein-phosphatase-1 and regulation of protein-kinase A activity (Fienberg et al. 1998; Svenningsson et al. 2004; Girault and Nairn 2021). In GMs, DARPP-32-immunoreactivity was detected in the NC, Pu, Acb, and the Tu, demonstrating a strong presence of post-synaptic dopaminergic elements in these structures.

Notably, DARPP-32-labeled sections of the Acb displayed patchy zones of strong immunoreactivity interspersed with less reactive regions. A similar, albeit distinct, patch pattern was also observed in anti-ChAT-stained sections. These patches likely resemble the striosome (patch) and matrix compartments of the Acb, which are associated with fast and slow dopamine kinetics, respectively (Jaquins-Gerstl et al. 2021). These compartmental patterns have been reported in rodents, cats, rhesus monkeys, and humans (Graybiel and Ragsdale 1978), and are thought to correspond to different areas of the SNc and VTA. Their presence in the GM Acb supports the existence of functional subregions and aligns with the distinct connectivity profiles observed in our tracing data. Additionally, the gradual anatomical transition from Acb to NC and Pu in the GM, rather than a sharply defined border as seen in rodents, supports a closer cytoarchitectural resemblance to primates.

ChATab marks cholinergic interneurons, which are tonically active and play essential roles in motor function, arousal, sleep, memory, and learning (Castro and Bruchas 2019). ChAT-positive neurons were present in the Acb, Sep, GP, NC, Pu, VP, and Tu, comparable to the distribution described in the human (Sutoo et al. 1994; Oda 1999), although the diagonal bands of Broca were not thoroughly analyzed.

TH, a marker of catecholaminergic neurons, was used to visualize presynaptic dopaminergic elements originating from the SNc and VTA. The TH-expressing cell population of the VS/VC is thought to act as local interneurons (Castro and Bruchas 2019) in the nigrostriatal and mesolimbic pathway, which originates from the SNc and the VTA, respectively. High TH-immunoreactivity is seen in the Acb, Amyg, mPFC, NC, Pu, and the Tu in both rodents and humans (Sutoo et al. 1994, 2002). In GMs, we observed strong TH immunoreactivity in the Tu, NC, and Pu, while the Acb displayed only weak TH staining, localized to the medial shell. This, in combination with robust DARPP-32 reactivity, suggests that the Acb of GMs primarily contains postsynaptic dopaminergic elements, whereas the Tu, NC, and Pu contain both pre- and postsynaptic dopaminergic components. However, this interpretation requires further confirmation.

SP, a neuropeptide expressed in D1-receptor-expressing medium spiny neurons (D1-MSN), marks the projection neurons in the direct pathway of striatal structures in the rat and human brain (Blomeley et al. 2009; Macpherson and Hikida 2019; Castro and Bruchas 2019). SP has been demonstrated to modulate stress-related anxiety and depressive behavior in rats through striatal stimuli (Husum et al. 2008; Iftikhar et al. 2020). We found anti-SP-positive elements in the NC, Pu, Acb, and Tu, indicating the presence of D1-MSNs within these regions in GMs, in line with data from rodents and humans.

The calcium-binding protein, CB, highly expressed in striatal tissue, has previously been used along with anti-SP antibodies to mark the CB-poor and SP-rich crescent-shaped shell compartment of the Acb, which is visible microscopically in rodents and on a molecular level in non-human primates and humans (Jongen-Rêlo et al. 1994; Meredith et al. 1996; Prensa et al. 2003). While both CB and SP were expressed in the GM Acb, a clear shell/core boundary could not be identified in the present study using these markers. This may indicate a more gradual or diffuse compartmental organization, which is broadly consistent with reports in primate and human, where the shell/core distinction is often subtle and sometimes detectable only at the molecular level. In a previous study from our group, the Acb could be subdivided into shell and core using anti-CB immunostaining; however, the boundary was likewise not sharply demarcated (Meidahl et al. 2016).

Lastly, MBP, a major structural component of myelin (Delassalle et al. 1981; Nabizadeh 2025), was used to visualize differences in white and grey matter within the VS/VC. As expected, MBP immunoreactivity was observed in white matter structures, including the aca, IC, EC, TWM, and corpus callosum, akin to data from other species.

In summary, the chemical and topographical organization of the GM VS/VC shows several similarities to that described in non-human primates and humans. The VP, although positioned more laterally in GMs than in humans, appears to exhibit broadly comparable connectivity patterns to those reported in human, macaque, and rodent studies. The relatively subtle compartmentalization of the Acb into shell and core may reassemble aspects of primate cytoarchitecture more than that typically observed in rodents. While the topographic organization of the GM VS/VC differs from that of rodents, key features of connectivity and neurochemical profiles appear to be generally conserved across species. Together, these observations suggest that the GM may represent a useful large animal model for investigating basal forebrain circuitry, with potential relevance to studies of reward processing, motivation, and neuropsychiatric disorders.

### Methodological considerations

Our multimodal approach, combining immunohistochemistry with six neurochemical markers, provides a neuroanatomical characterization of the GM VS/VC. Although the sample size was limited, the findings are generally consistent with existing descriptions and available atlases of the GM brain (Bjarkam et al. 2017a; Orlowski et al. 2019). All animals survived the 28-day tracer distribution period. Neuronal tracing is an established method to reveal neuronal connectivity, and MRI coordinates used for tracer injection target were obtained with millimeter precision, confirmed by post-mortem microscopy. Despite these measures, some methodological limitations must be acknowledged. All GMs displayed some degree of tracer reflux, mainly within the striatum. In particular, microscopic examination of tissue along the injection tracts, above the target, revealed low/very low BDA uptake by neuronal somata in brains AVS1 and AVS4, limited uptake in the striatum/internal capsule region in AVS2, and some uptake mainly just above the injection site along the internal capsule in AVS3. Therefore, even though tracer uptake in target areas was much larger than above them, we cannot exclude the possibility that some tracing results may relate to the tracer uptake occurring from the cells lying outside the injection target or from the fibers of passage. In the case of FG sections, labeling was also observed along the injection track above the target region in a similar pattern as in the case of BDA. This introduces the possibility of some false-positive labeling in structures outside the intended injection site, particularly in proximity to the aca. Conversely, negative findings may reflect either limited tracer distribution, especially in the case of BDA, or a true absence of projections. Thus, the connectivity profile presented here should be interpreted with caution. Integration of MRI-based tractography, as applied in our previous studies (Bech et al. 2018, 2020) would provide valuable complementary validation. Likewise, the sectioning interval (800 μm) used in this study may have limited the detection of small or spatially restricted projections, and together with the small number of animals and the technical challenges associated with aligning large brain sections, makes it difficult to reconstruct the three-dimensional extent of tracer labeling or to perform stereological quantitative analyses.

Our neurotransmitter analysis employed markers such as DARPP-32, ChAT, and SP, but did not directly assess GABAergic, glutamatergic, enkephalin, or serotonergic systems, neurotransmitter networks critically involved in basal ganglia function and mood regulation (Gabbott et al. 2005). Future studies should expand the neurochemical profiling of the GM VS/VC using these markers and employ viral or genetically encoded tracers to improve connectional specificity. Another limitation of our study was the exclusive use of young female animals. While including males would strengthen our findings, technical constraints made this unfeasible, since male pigs are more aggressive and require separate housing. Furthermore, bladder catheterization, necessary during prolonged surgical procedures, is very challenging or impossible in males (Ettrup et al. 2011). Similarly, restricting the tracing experiments to the right hemisphere may be a limitation. Recent studies have described structural asymmetries in the porcine brain, which could potentially result in interhemispheric differences in connectivity patterns (Fujiwara et al. 2026). MDD is a complex, multifactorial condition, and DBS efficacy in TRD may likely involve the modulation of multiple interconnected targets (Runia et al. 2025; Mol et al. 2025). The present findings suggest that the GM VS/VC shares several anatomical and neurochemical features with primates, including substantial connectivity with cortical, limbic, and brainstem structures. These observations indicate that the GM may represent a useful large-animal model for studying DBS-related mechanisms, exploring electrode placement, and examining stimulation parameters, as well as for the development of electrophysiological biomarkers for closed-loop neuromodulation. Overall, our results contribute to a more detailed understanding of GM VS/VC organization and connectivity and may provide a foundation for future preclinical studies aimed at evaluating potential DBS targets and stimulation strategies, with possible relevance for translational research in treatment-resistant depression.

## Conclusion

Our findings indicate that the GM VS/VC shares several structural and connectional features with both rodent and primate counterparts. These observations suggest that the Göttingen minipig could serve as a useful large-animal model for investigating the effects of DBS and other neuromodulatory interventions. While limitations such as the small sample size and the potential influence of fibers of passage should be considered when interpreting data, the results presented here contribute to the growing anatomical knowledge of the VS/VC region and may provide a basis for future studies exploring neuromodulation in a translational context.

## Data Availability

No datasets were generated or analysed during the current study.

## Abbreviations

ABC: Avidin-Biotin Complex Vectastain kit
aca: Anterior (ventral) crus of the anterior commissure
Acb: Nucleus accumbens
acp: Posterior (dorsal) crus of the anterior commissure
ALIC: Anterior limb of the internal capsule
Amyg: Amygdala
BDA: Biotinylated dextran amine
CB: Calbindin D-28k
ChAT: Choline acetyltransferase
D1-MSN: Dopamine-receptor D1-expressing medium spiny neuron
DARPP-32: Dopamine- and cAMP-regulated phosphoprotein,32kDa
DAB: 3,3’-diaminobenzidine tetrahydrochloride
DBS: Deep-brain stimulation
EC: External capsule
FG: FluoroGold
GM: Göttingen minipig
GP: Globus Pallidus
HLDB: Horizontal limb of the diagonal band
Hip: Hippocampus
HRP: Horseradish peroxidase
IC: Internal capsule
ir: immunoreactive
LO: Lateral olfactory tract
mAb: Monoclonal antibody
MBP: Myelin basic protein
MDD: Major depressive disorder
MSC: Medial septal complex
mPFC: Medial prefrontal cortex
NC: Caudate nucleus
OB: Olfactory bulb
OCD: Obsessive-compulsive disorder
pAb: Polyclonal antibody
PAG: Periaqueductal gray matter
PB: Phosphate buffer (pH 7.4)
PBS: Phosphate-buffered saline (pH 7.4)
PFA: 4% Phosphate-buffered formaldehyde (pH 7.4)
PrPir: Prepiriform cortex
Pu: Putamen
SCA: Subcallosal cingulate area
Sep: Septum
SNc: Substantia nigra pars compacta
SP: Substance P
TBS-T: Tris-buffered saline (0.05 M, pH 7.4) + 1% Triton X-100
TH: Tyrosine hydroxylase
Thal: Thalamus
TRD: Treatment-resistant depression
Tu: Olfactory tubercle
TWM: Telencephalic white matter (homolog to centrum semiovale, cso)
VC: Ventral capsule
VLDB: Ventral limb of the diagonal band
vHip: Ventral hippocampus
VP: Ventral pallidum
VS: Ventral striatum
VTA: Ventral tegmental area
